# Investigation of the Microstructure and Mechanical Properties of Heat-Treatment-Free Die-Casting Aluminum Alloys Through the Control of Laser Oscillation Amplitude

**DOI:** 10.3390/ma18061194

**Published:** 2025-03-07

**Authors:** Hong Xu, Jinyi Shao, Lijun Han, Rui Wang, Zhigong Jiang, Guanghui Miao, Zhonghao Zhang, Xiuming Cheng, Ming Bai

**Affiliations:** 1 Key Laboratory of Automotive Materials Ministry of Education, School of Material Science and Technology, Jilin University, Changchun 130022, China; 2 College of Materials Science and Engineering, Jilin University, Changchun 130025, China; 3 FAW-Volkswagen Automotive Co., Ltd., Changchun 130011, China; 4 CCAS (Changchun) Steel Service Center Ltd., Changchun 130012, China; 5 North Mechanical and Electrical Co., Ltd., Shijiazhuang 050800, China

**Keywords:** AlSi7MnMg die-casting aluminum alloy, laser oscillation, molten pool flow, microstructure

## Abstract

In this study, laser oscillation welding was utilized to offer an effective solution for the joint welding of heat-treatment-free die-cast aluminum alloys, which expands the practical applications of automotive structural parts and heat sinks for electronic devices. The effects of oscillation amplitude on the macro-morphology, microstructure, and properties of the alloy weld were examined, and a molten pool flow model was developed to compare the behavior of the molten pool with and without oscillation. The results show that increasing the oscillation amplitude eliminates the coarse Al_15_(Fe,Mn)_3_Si_2_ phase, resulting in a finer and more uniform distribution of the eutectic Si and Mg_2_Si phases. At an oscillation amplitude of 7 mm, the maximum tensile shear load and displacement were 2761 N and 1.17 mm, respectively. Laser oscillation was found to enhance the fluidity of the molten pool, reduce porosity, improve weld quality, and effectively decrease cracks and inhomogeneous grain distribution. These findings provide a research basis for optimizing the laser oscillation welding process and for the practical welding of fabricated devices.

## 1. Introduction

In recent years, die-casting aluminum alloys have been widely used in complex parts for new energy vehicles, cell phone radiators in the electronics industry, aircraft engine components, and more due to their excellent casting properties, high specific strength, and superior electrical and thermal conductivity [1,2,3,4,5]. The material analyzed in this study is AlSi7MnMg, a heat-treatment-free die-cast aluminum alloy. This material not only shares similar welding challenges with extruded aluminum alloys [6], such as the tendency to generate porosity, cracks, and humping [7,8,9], but also faces increased welding complexity due to the high hydrogen content [10] and shrinkage defects inherent in die-cast parts [11].

Conventional welding techniques, such as tungsten inert gas (TIG) welding [12] and friction welding [13], often encounter difficulties when welding aluminum alloys. Aluminum alloys are prone to forming a dense oxide film in air and absorbing moisture, which can lead to defects such as slag inclusion, cracks, and porosity during the welding process [14,15,16,17]. Furthermore, die-casting aluminum alloys have a significantly higher hydrogen content than conventional aluminum alloys. This makes it difficult for hydrogen to escape during the melting process, resulting in porosity formation [18].

To address these issues, laser welding technology has been widely adopted. Laser, as a heat source, provides high energy density [19,20], concentrated heat input [18,21], and a rapid welding process. These factors contribute to a narrower heat-affected zone, smaller deformation, higher control precision, and improved welding efficiency. Compared to conventional welding methods, laser welding effectively enhances weld quality and minimizes defects. However, conventional laser welding of aluminum alloys still faces challenges. Due to the high hydrogen content and the rapid solidification of the molten pool during the laser welding process, hydrogen cannot escape in time, resulting in the formation of porosity [22].

Currently, extensive research has been conducted both domestically and internationally on new laser welding methods. For conventional laser welding technology, the introduction of linear oscillation has been studied in terms of its impact on weld morphology, microstructure, and mechanical properties. This research has clarified the mechanism by which linear oscillation alters the distribution of heat input in the molten pool and the direction of the temperature gradient, promoting the formation of fine grains. Li et al. [23] demonstrated that when the oscillation frequency exceeds 200 Hz and the oscillation diameter is greater than 2 mm, laser beam oscillation can reduce weld porosity, refine the grain structure, and promote the uniform distribution of the β(Mg_2_Al_3_) phase in the weld fusion zone. Cheng et al. [24] investigated the regular changes in weld morphology and microstructure during the oscillating laser welding of 2219 Al-6Cu alloy under varying power and amplitude, identifying three interaction modes between the laser and material: keyhole, transition, and conduction modes. Cen et al. [25] found that the rapid periodic motion of high-frequency oscillating lasers facilitated a uniform distribution of melt energy, enhanced melt flow, and transformed the intergranular precipitation phase from a coarse continuous line to a spider-web structure. Mohan et al. [26] examined the effect of laser welding solidification parameters on microstructure formation, with finite element simulations showing that the cooling rate increased with higher oscillation frequencies. Zhou et al. [27] applied four different types of laser oscillation trajectories (linear, ∞-shaped, figure-eight-shaped, and conventional straight lines) to the welding of 2060 aluminum alloy, finding that oscillating lasers resulted in better bending resistance, tensile properties, and microhardness—as well as finer grains—when compared to conventional laser joints. Meng et al. [28] used figure-eight beam oscillations to address the issues of inhomogeneous interface heating and reactions in Al/Mg lap joints with titanium as the intermediate layer during laser ring oscillation welding.

At present, research on laser oscillatory welding of heat-treatment-free die-cast aluminum alloy materials is limited, and the underlying mechanism is not clear. Cai et al. [29] improved energy distribution, reduced pore size, improved grain refinement, and enhanced the mechanical properties of 5A06-H112 aluminum alloy by varying the amplitude (1–3 mm) and frequency (100–300 Hz) of the clockwise circular oscillations. Jiang et al. [30] employed laser oscillation welding to weld Invar alloy, adjusting the frequency and amplitude of the beam oscillation to achieve grain refinement and reduce internal defects, with the selected amplitudes being 0, 2, 4, and 6 mm. The aim of this study is to verify the feasibility of aluminum-silicon system die-cast aluminum alloys in laser oscillatory welding. To achieve this, oscillatory laser welding experiments were conducted, and the laser oscillation amplitude was adjusted to 4, 5, 6, and 7 mm while keeping other welding parameters constant. Then, the effects of laser oscillation on weld morphology, microstructure, and mechanical properties were investigated.

## 2. Materials and Methods

The test materials were AlSi7MnMg vacuum die-cast plates with 2.0 mm and 3.0 mm thicknesses, which were used to prepare lap fillet weld laser specimens. The chemical composition of the raw materials is presented in Table 1.

**Figure 1:**
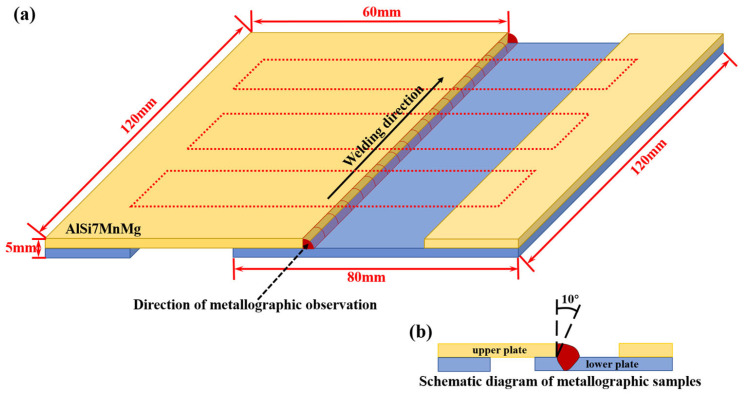
(**a**) Position of plate lap welding; (**b**) schematic diagram of metallographic samples.

This study investigates the effect of laser oscillation amplitude on weld seam morphology under a linear oscillation trajectory. The experimental parameters are shown in Table 2, with an oscillation frequency of 100 Hz and amplitudes of 0, 4, 5, 6, and 7 mm. As shown in Figure 1a, butt-joint (angle-shaped) laser welding was performed, with the upper plate measuring 60 mm × 120 mm × 2 mm and the lower plate measuring 80 mm × 120 mm × 3 mm. The overlap of the joint was 30 mm. To address surface defects caused by non-polishing and to enhance welding performance [31], the oxide film on the substrate surface was removed using sandpaper and a steel brush prior to laser welding; the surface was cleaned with acetone to remove oil contamination. The laser used in the welding process was the Laserline LDF 4000-6 diode laser (Laserline GmbH, Puchheim, Germany) capable of outputting up to 4000 W of power, with a voltage of 400 V and a frequency of 50/60 Hz. After welding, the samples were taken for metallographic analysis, with the sampling location and observation direction shown in Figure 1b. The cross-sectional morphology and microstructure of the weld seam were observed using optical microscopy (OM) and scanning electron microscopy (SEM). Energy dispersive spectroscopy (EDS) and X-ray diffraction (XRD) were employed for elemental and physical analysis of the weld seam, respectively. In addition, shear tests were conducted at room temperature using the Shimadzu AG-IS universal material testing machine (Shimadzu, Kyoto, Japan).

## 3. Results and Discussion

### 3.1. Weld Morphology of AlSi7MnMg Lap Joints with Different Laser Oscillation Amplitudes

#### 3.1.1. Effect of Laser Oscillation Amplitude on the Macroscopic Morphology of the Weld Seam

Oscillation amplitude refers to the maximum distance that the laser spot moves away from the weld center on both sides in the vertical welding direction. It is a crucial parameter in laser oscillation welding. At the same oscillation frequency, a larger oscillation amplitude results in a broader laser spot area, which subsequently reduces the heat input. Additionally, the thermal action time on the weld varies with the amplitude, altering the laser beam’s speed and thereby influencing the keyhole dynamics and the molten pool condition.

**Figure 2:**
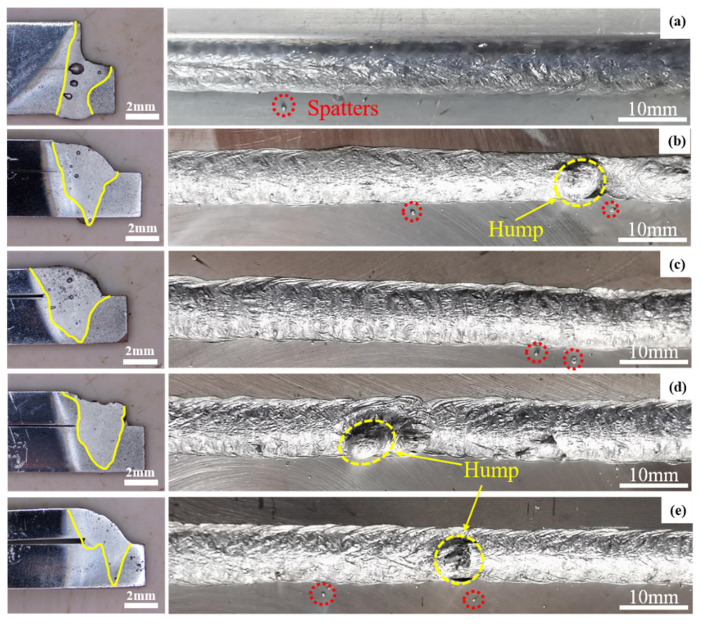
Surface morphology of welded samples at 100 Hz for (**a**) 0 mm, (**b**) 4 mm, (**c**) 5 mm, (**d**) 6 mm, and (**e**) 7 mm amplitudes, respectively.

As shown in Figure 2, the cross-sectional and surface morphology of the welds are presented for oscillation frequencies of 100 Hz and oscillation amplitudes ranging from 4 mm to 7 mm. In conventional laser welding, the pore size is larger, whereas in oscillated laser welding, the pore size is smaller. The weld under non-oscillated conditions exhibits a concave weld angle, while after oscillation, the weld angle becomes convex. As shown in Figure 2, the cross-sectional and surface morphology of the welds are presented for oscillation frequencies of 100 Hz and oscillation amplitudes ranging from 4 mm to 7 mm. In conventional laser welding, the pore size is larger, whereas in oscillated laser welding, the pore size is smaller. The weld under non-oscillated conditions exhibits a concave weld angle, while after oscillation, the weld angle becomes convex. A hump appears at the sampling location in the weld with a 6 mm amplitude, and a distinct serrated structure is observed in the sample with a 7 mm amplitude. Since the energy distribution in conventional laser welding follows a normal distribution, the heat at the center of the molten pool is higher than that at the edges, leading to a concave shape at the pool’s center. The oscillation of the laser beam increases the time the molten pool remains in a liquid state, thus reducing the laser beam energy required for the next part of the melting metal. The metal melting is sufficient to produce an effective connection, and it is inferred that the welding quality after oscillation is superior.

It was also noted that varying degrees of spattering were observed at different oscillation amplitudes, and a hump formed after oscillation. The formation of humps during laser oscillation welding of aluminum alloys results from the application of linear oscillations, where the liquid metal is blocked by the sidewalls, and the laser beam moves back and forth. As a result, the liquid metal accumulates at the front of the molten pool instead of flowing smoothly to the rear. Additionally, liquid metal returning from the front meets liquid metal returning from the rear, forming a hump zone in the middle of the molten pool. This reduces the stability of the melt flow and creates a rough weld surface.

#### 3.1.2. Effect of Laser Oscillation Amplitude on Weld Seam Size

The thickness of the deep fusion weld (S) is an important parameter for assessing weld size, while the length of the weld feet (Z1, Z2) determines the cross-sectional area and geometry of the weld [32]; this, in turn, affects its performance, including strength, sealing, and appearance.

**Figure 3:**
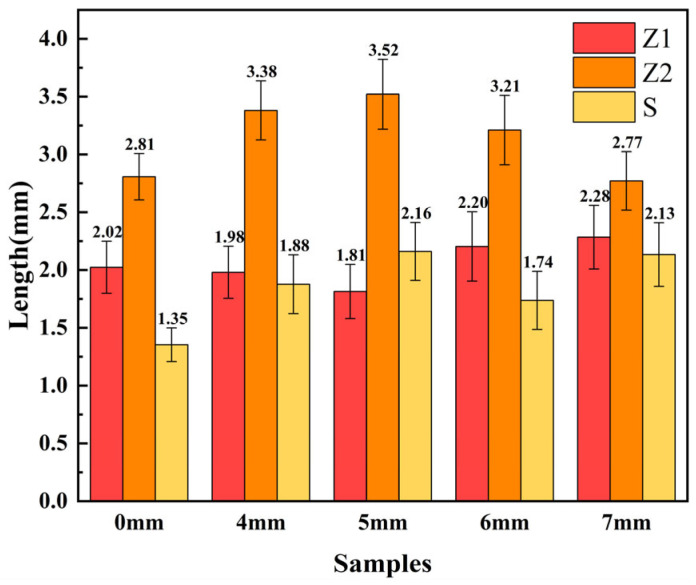
Statistical chart of Z1, Z2, and S for each weld cross-section with increasing laser oscillation amplitude.

As shown in Figure 3, the values of Z1, Z2, and S for each weld cross-section are plotted. With increasing laser oscillation amplitude, Z1 tends to decrease first and then increase, while Z2 shows the opposite trend, increasing and then decreasing. The decrease in the Z1 value at an oscillation amplitude of 5 mm compared to 4 mm is due to the increased overlap between the laser scanning lines as the oscillation amplitude increases, causing a faster molten pool flow in the middle of the weld, resulting in a reduction in Z1. As the amplitude increases further, Z1 begins to rise because the wider scanning amplitude increases the width of the molten pool and expands the heat-affected area on the upper plate, leading to more metal melting in the upper plate and, consequently, a larger Z1 value.

When the amplitude increases from 4 mm to 5 mm, the Z2 value shows a slight increase. This is because the increase in amplitude causes only a small reduction in heat input, and the laser scanning area expands, directly increasing the Z2 value. However, as the amplitude continues to increase, the heat input per unit area decreases, leading to a reduction in the Z2 value.

As shown in Table 3, the variation in weld foot length (ΔZ) initially increases and then decreases with the rise in oscillating welding amplitude. The ΔZ value reaches its peak of 1.71 mm at an amplitude of 5 mm, but when the amplitude increases to 7 mm, the ΔZ value drops to 0.49 mm. This suggests that the sample welded at an amplitude of 7 mm exhibits the highest weld uniformity, which is likely to result in superior mechanical properties.

#### 3.1.3. Effect of Laser Oscillation Amplitude on Reynolds Number

To assess the flow state of liquid metal in the molten pool, it is essential to calculate the Reynolds number [33], which describes the relative importance of inertial and viscous forces in the fluid. The Reynolds number is calculated as follows [34]:

(1) $$Re=\frac{\rho ND^{2}}{\mu }=\frac{\rho f{\left(2A\right)}^{2}}{\mu }$$

**Figure 4:**
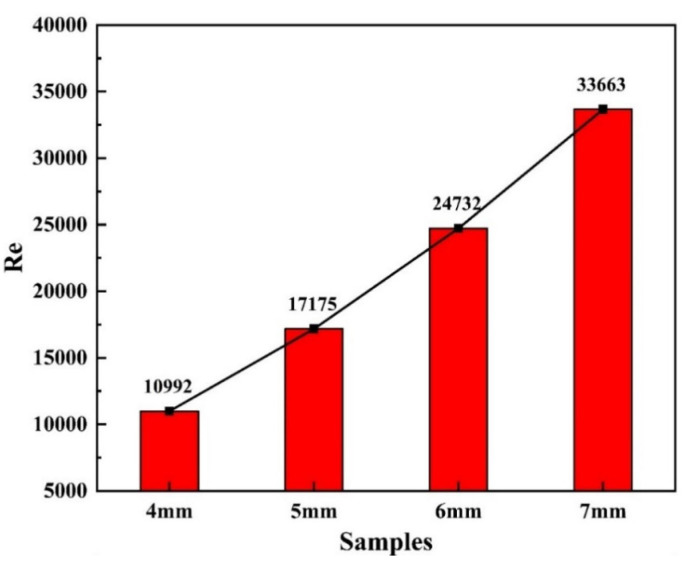
Reynolds number of samples welded using oscillating laser.

In the equation, *Re* is the Reynolds number, *ρ* is the density of the molten pool, *μ* is the viscosity of the melt flow, and their values are 2370 kg/mm^3^ and 1.38 × 10^−3^ Pa·s, respectively [34]; *f* and *A* are the frequency and amplitude of the laser oscillation welding, respectively. The Reynolds number of the molten pool with different oscillation amplitudes can be calculated according to an equation (Figure 4) so as to determine whether the flow state of the fluid is turbulent (*Re* > 1000) or laminar (*Re* < 1000). It was observed that the amplitude, A, in this experiment was large, resulting in Reynolds numbers greater than 1000 for all samples, indicating turbulent flow in the molten pool. The intensification of turbulence promotes the eruption of metal vapor, leading to spattering in the weld seam. At the same time, the increased turbulence enhances convective heat transfer within the molten pool, particularly causing heat accumulation at the bottom of the pool. This heat accumulation makes the liquid metal at the bottom of the molten pool prone to sagging due to insufficient gravitational and surface tension forces, thereby directly facilitating the formation of humping. Moreover, turbulence may alter the flow patterns of the liquid metal within the molten pool, further exacerbating the sagging tendency and increasing the likelihood of hump formation.

#### 3.1.4. Influence of Laser Oscillations on the Flow State of the Weld Pool

During the formation of the molten pool, interactions between its surface and the surrounding environment cause unstable bumps and depressions, manifesting as surge phenomena. This behavior is primarily due to the relatively high thermal conductivity of aluminum alloys, which causes rapid heat dissipation after heating and results in pronounced surge effects in the molten pool. When the laser beam is focused on the surface of the workpiece, the absorbed laser energy creates a high-temperature region, forming a small molten pool. Due to aluminum’s high thermal conductivity, the molten pool quickly spreads, while new molten pools continue to form as the laser beam progresses.

**Figure 5:**
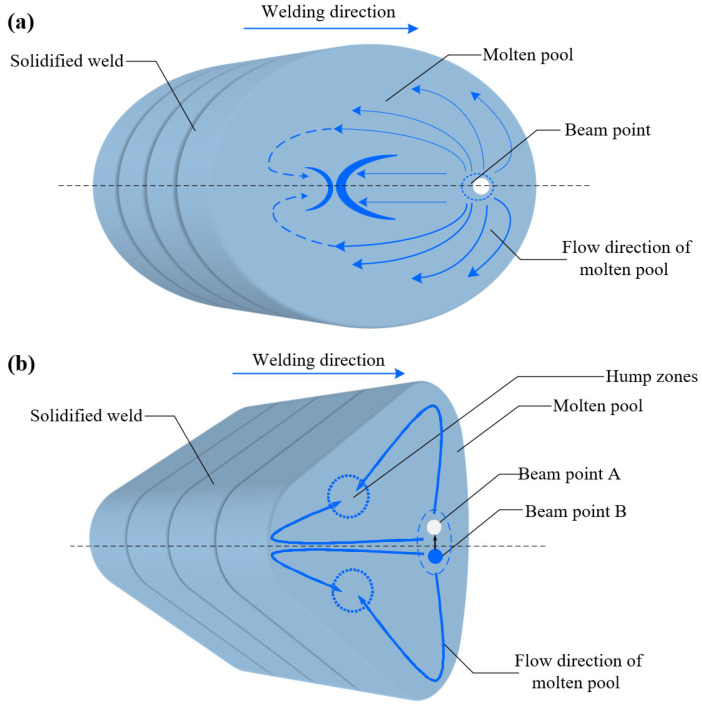
(**a**) Schematic diagram of the surface flow behavior of a regular laser and (**b**) a linear oscillating welding pool.

Figure 5a shows that as the keyhole advances in the welding direction, the molten pool also moves forward. The surface tension of the molten pool and the inertia of the melt generate a surge that flows in the opposite direction to the weld. When this surge encounters the rear of the molten pool, a foldback surge occurs. However, since part of the driving energy is lost during the collision, only a small surge folds back, which is subsequently covered by the next wave moving rearward. This regular flow behavior on the molten pool’s surface produces a rounded and orderly fish-scale pattern. However, since the surge behavior is not strictly periodic, the spacing of the fish scales is uneven, as shown in Figure 2. As shown in Figure 5b, when linear oscillations were applied, the area of the molten pool increased only slightly compared to conventional laser welding, but its shape transformed into a teardrop, wider at the front and narrower at the rear. As the laser moves downward along its path, the molten pool is influenced by the stirring action of the keyhole, generating a surge that flows downward and to the left. Due to the characteristic lag of the surge behind the movement of the laser and keyhole, the surge does not reach the lowest part of the molten pool by the time the keyhole moves to the foldback point. Additionally, because of the high oscillation frequency, the surge created during the upward movement of the previous laser cycle is still moving toward the upper-left boundary of the molten pool, creating a flow along the pool boundary and toward the foldback point.

When the laser moves upward along the action track, the molten pool is, again, influenced by the stirring effect of the keyhole, generating a surge that flows to the upper left. As before, the surge lags behind the movement of the laser and keyhole, and it does not reach the bottom of the molten pool when the keyhole reaches the foldback point. Furthermore, the surge from the previous downward laser movement flows toward the lower-left boundary of the molten pool, producing a small surge along the boundary and toward the foldback point. This cyclical flow behavior on the molten pool’s surface corresponds to the oscillation cycle. During each oscillation cycle, two surges move in a direction similar to that of the keyhole motion. However, because this direction forms a small angle with the keyhole’s path, the flow differs from that seen in conventional laser welding, significantly affecting the molten pool’s shape on one side. This results in a fish-scale pattern that is not a half-arc but rather a quarter-arc.

When the fast-moving surge encounters a particle or void, significant fluctuations occur. At this point, if the returning smaller surge coincides with the large fluctuation, welding defects such as humps and spatter are likely to form. It is expected that the next phase of the study will further investigate the molten pool flow process using a high-speed camera. This will involve combining the experimentally measured molten pool geometry, flow characteristics, and other data and comparing them with the predicted results from the molten pool flow model. This approach will enable a comprehensive verification of the model’s accuracy.

### 3.2. Effect of Laser Oscillation Amplitude on the Microstructure of Welded Joints

#### 3.2.1. Grain Characteristics of Welded Joints

**Figure 6:**
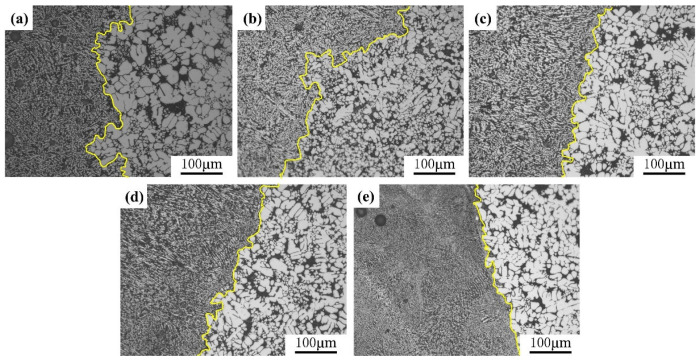
When the frequency is 100 Hz, the OM microstructure diagram of each joint position is as follows: (**a**) 0 mm, (**b**) 4 mm, (**c**) 5 mm, (**d**) 6 mm, and (**e**) 7 mm.

The optical microscopy (OM) microstructure of each joint position is shown in Figure 6. The boundary of the fusion zone (yellow lines) becomes progressively flatter with the oscillation amplitude. This is due to the increased molten pool area and accelerated flow rate under oscillation, which results in a smoother interface. At a constant frequency of 100 Hz, the increase in amplitude means that the higher the longitudinal oscillation speed of the laser beam, the shorter the steering time of the laser beam at both ends; there is a point where there is a speed of zero at both ends, where the heat input is reduced, leading to a gradual refinement of the grains at the boundary of the fusion zone. As shown in Figure 6c,d, the weld exhibits a vertically arranged mesh-like dendritic structure known as shear dendrite. This occurs because, at the weld boundary, a large temperature gradient along the fusion line results in a slow crystallization rate, and supercooling makes it difficult for the composition to form. As the grains grow from the boundary towards the weld center, the temperature gradient gradually decreases, leading to an accelerated crystallization rate. The mass fraction of solute increases, and the supercooled region also gradually expands. The sub-structure within the columnar crystals evolves the columnar crystal within the sub-structure to cellular crystals, cellular dendritic crystals, and the development of shear dendritic crystals. The presence of these shear dendritic structures causes stress concentration in the weld, becoming the initiation points for crack formation and propagation. Additionally, these dendrites reduce the plasticity of the weld, negatively affecting its impact and fatigue resistance. As shown in Figure 6e, remelting and delamination occur when the amplitude increases to 7 mm. This phenomenon arises because an excessive amplitude amplifies the temperature gradient in the weld area, causing thermal effects to become uneven.

#### 3.2.2. Second Phase Distribution

**Figure 7:**
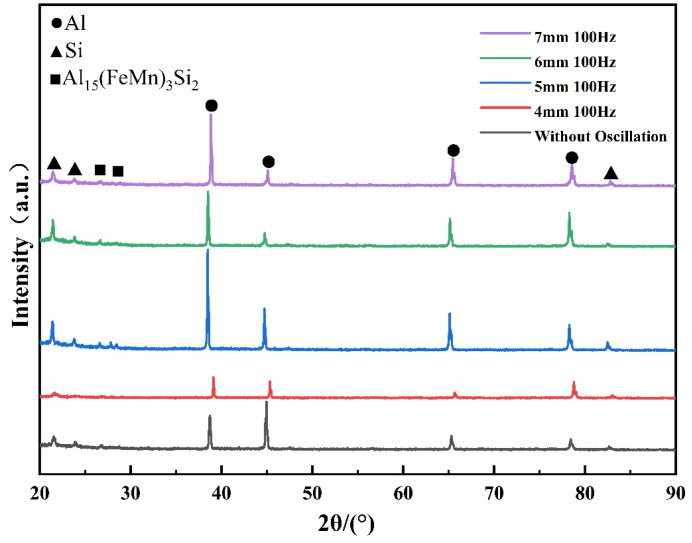
XRD diagrams of welded samples at different laser amplitudes at 100 Hz.

**Figure 8:**
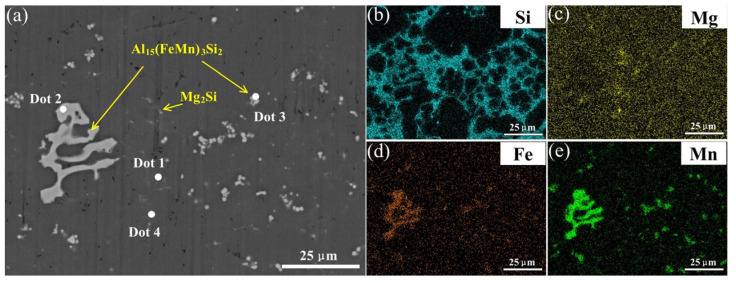
(**a**) shows the backscattered electron map of the parent material; (**b**–**e**) show the energy spectra of the elements.

Figure 7 presents the results of X-ray diffraction (XRD) analyses for all welded joints, showing that the diffraction peak of the Si phase decreases as the oscillation amplitude increases. These results suggest that the phase type remains unchanged by the welding process and variations in laser oscillation amplitude. Combined with the backscattered electron scanning electron microscopy (BSE) and energy-dispersive spectroscopy (EDS) analyses shown in Figure 8, the samples primarily contain the eutectic Si phase, an AlFeMnSi phase, and an MgSi phase in addition to the α-Al phase. The eutectic Si phase is mainly located between the Al grains, while the AlFeMnSi phase exhibits two primary morphologies: one is iso-axial particles with regular shapes, uniformly dispersed along the grain boundaries; the other is a coral-like phase with larger, irregularly shaped particles. The MgSi phase appears in rod-like forms. To further determine the chemical composition, the positions marked in Figure 8 were subjected to elemental analysis, as shown in Table 4. According to the elemental analysis results, the AlFeMnSi phase is presumed to be the Al_15_(Fe, Mn)_3_Si_2_ phase, where some Fe atoms replace Mn atoms during the phase formation process. This explains why the distribution maps of Mn and Fe elements are almost identical; thus, the Mn element distribution map is used in place of the Fe element distribution map. The Mn element distribution map effectively represents both Fe and Mn elements. Additionally, the MgSi phase corresponds to the Mg_2_Si phase, which forms through a eutectic reaction: $l\to \alpha -Al+\beta -Si+{Mg}_{2}Si$ during the solidification process.

**Figure 9:**
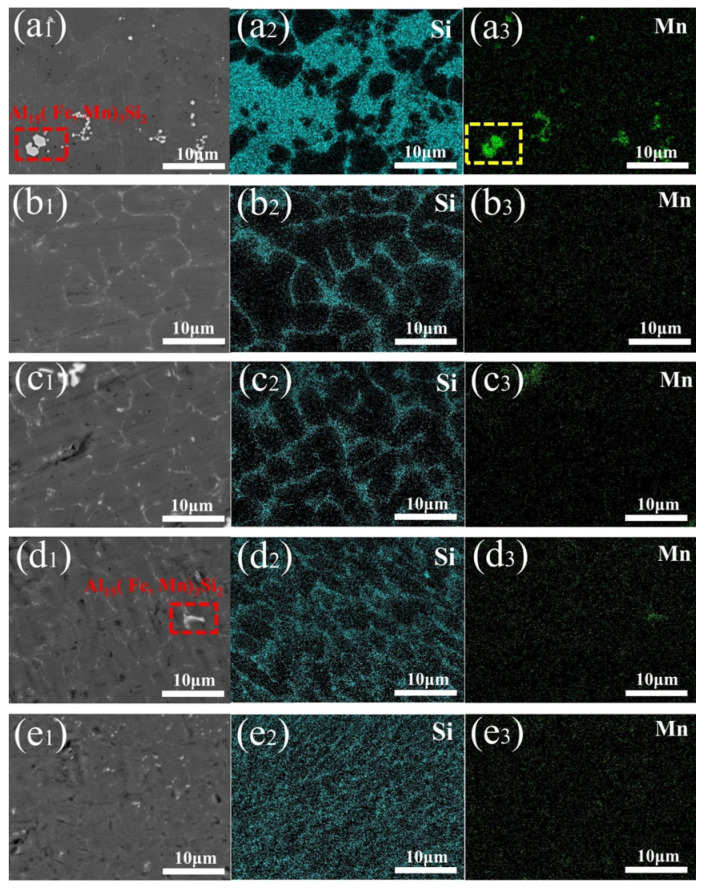
BSE-EDS diagrams of welded joints at 100 Hz: (**a_1_**–**a_3_**) 0 mm, (**b_1_**–**b_3_**) 4 mm, (**c_1_**–**c_3_**) 5 mm, (**d_1_**–**d_3_**) 6 mm, and (**e_1_**–**e_3_**) 7 mm.

The BSE-EDS images of welded joints with different oscillation amplitudes are shown in Figure 9. Figure 9a_1_–e_1_ show that with increasing amplitude, the fluidity of the molten pool is enhanced, which leads to the gradual disappearance of the blocky Al_15_(Fe, Mn)_3_Si_2_ phase (red dashed squares) and block shaped Mn elements (yellow dashed squares). The formation of the Mg_2_Si phase is dependent on the chemical reaction between the Mg and Si elements in the alloy, as well as their diffusion behavior during the welding process. With the change in oscillation amplitude, the welded joint moves constantly, the heat input is more uniform, and cellular crystals and dendrites are generated; this organization is conducive to the uniform distribution of the Mg_2_Si phase. A uniformly dispersed Mg₂Si phase can refine the grain, improve the hardness of the material, and, thus, enhance the mechanical properties of the welded joint [35].

Compared to the α-Al phase, the eutectic Si phase is known to be hard and brittle. As a result, the coarse eutectic silicon phase and its uneven distribution are considered key factors that hinder the strength and plasticity of the alloy. It has been demonstrated by Xu et al. [36] that beneficial changes in the eutectic silicon structure contribute to the improved tensile properties of the A356 alloy. Figure 9a_2_–e_2_ illustrate how laser oscillation modifies the distribution of silicon elements. In conventional laser welding, the eutectic Si phase tends to aggregate and form flake-like structures. With the introduction of laser oscillation, the uneven distribution of silicon is improved, and the area occupied by the eutectic silicon phase gradually decreases. As the oscillation amplitude increases, the broad aggregation of silicon elements is broken, and the eutectic phase begins to form a network-like distribution along the grain boundaries. By using an amplitude of 7 mm, the eutectic Si phase is seen to be diffusely distributed.

#### 3.2.3. Effect of Different Oscillation Amplitudes on Pulling Shear Performance

**Figure 10:**
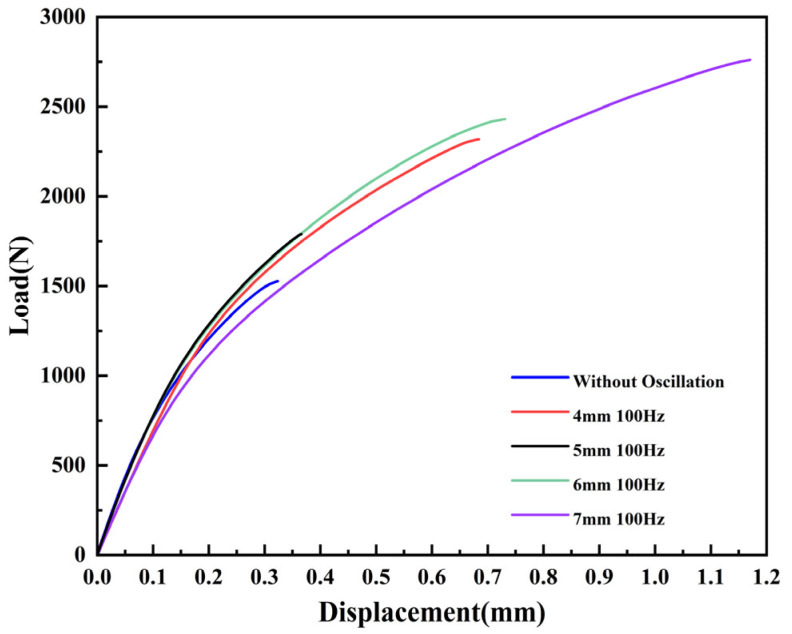
Tension-shear curves of welding samples under different laser oscillation amplitudes at 100 Hz frequency.

The corresponding tensile test results are shown in Figure 10, where it is observed that the tensile shear load and displacement of the oscillated samples are both higher than those of the non-oscillated samples. As shown in Table 5, when the oscillation parameters are set to 7 mm/100 Hz, the best performance is achieved, with the maximum tensile shear load and displacement increasing to 2761 N and 1.17 mm, respectively. These values are 80.9% and 254.5% higher than those of the non-oscillated laser welding. During the beam oscillation process, the grain size of the weld is refined due to preheating and remelting, and the tensile test further confirms that the mechanical strength of the material is significantly improved by laser oscillation. The enhancement of mechanical properties is not only due to grain size refinement but also results from the reduction in porosity and the uniform distribution of blocky Al_15_(Fe, Mn)_3_Si_2_ and Mg₂Si phases and eutectic Si phases during the laser beam oscillation process, which also contributes to the improvement of tensile performance.

## 4. Conclusions

This paper verifies the feasibility of oscillating laser welding technology to improve the welding quality of die-cast aluminum alloys. Compared with traditional laser welding, oscillating laser welding has a greater potential in welding AlSi7MnMg die-cast aluminum alloy plates. The experiment helped to analyze the effect of changes in oscillation amplitude on the macroscopic morphology, microstructure, and properties of the joint. A molten pool flow model was established, which will be further investigated by simulating molten pool behavior. These findings provide valuable insights into further research on molten pool mechanisms in the laser oscillation welding of die-cast aluminum alloys. The main conclusions of this study are as follows:

1. Laser oscillation welding significantly improved the morphology of lap fillet welds in AISi7MnMg aluminum alloy plates. The collapse defect caused by conventional laser welding is avoided, and there is an obvious difference in the weld angle, which is transformed from a concave weld to a convex weld, effectively increasing the bonding area between the base materials. The increase in the bonding area is conducive to the stability of the welded structure and the improvement of the load-carrying capacity, and it is better to apply the laser oscillatory welding of AISi7MnMg aluminum alloy to the connection between the structural parts of new energy vehicles. In addition, the weld surface ripples changed from irregular arc ripples to more uniform quarter-arc ripples.
2. The laser oscillation welding process significantly improves the microstructure of the weld. During the oscillation process, the equiaxed grain zone in the fusion zone expands beyond the center of the weld. In conventional laser welding, a wide range of eutectic silicon phases tends to aggregate, forming a lamellar structure; with the addition of laser oscillation and amplitude increases, eutectic silicon aggregation is broken; a large area of continuous distribution is gradually transformed into a network along the grain boundaries of the distribution and is finally uniformly dispersed. Simultaneously, the fluidity of the molten pool has been effectively enhanced, leading to the gradual disappearance of the blocky Al_15_(Fe, Mn)_3_Si_2_ phase, whereas the quantity of the Mg_2_Si phase remains unchanged but is distributed more uniformly.
3. The laser oscillation process improves the mechanical properties of the lap fillet welds of non-heat-treated die-cast aluminum alloys. Compared to conventional laser-welded joints, oscillation laser-welded joints exhibit superior tensile shear properties. The optimal performance is achieved when the oscillation parameters are set at 7 mm/100 Hz, resulting in increases of 80.9% and 254.5% in maximum tensile shear load and displacement, respectively.

## Figures and Tables

**Table 1:** The measured compositions of AlSi7MnMg alloy.

Samples	Laser Power P (kW)	Welding Speed v (mm/s)	Oscillation Amplitude A (mm)	Oscillating Frequency f (Hz)
1	4	50	0	100
2			4	
3			5	
4			6	
5			7	

**Table 2:** Main experimental parameters.

Compositions	Si	Fe	Mn	Mg	Ti	Sr	Al
wt.%	7.12	0.13	0.61	0.21	0.05	0.008	balance

**Table 3:** Length difference, ΔZ, of each weld with different amplitudes.

Samples	Without Oscillation	4 mm/ 100 Hz	5 mm/ 100 Hz	6 mm/ 100 Hz	7 mm/ 100 Hz
ΔZ/mm	0.79	1.40	1.71	1.01	0.49

**Table 4:** EDS results of Dot 1–Dot 4.

Points	Atom Percent (at.%)					Possible Phases
	Al	Si	Mn	Fe	Mg	
Dot 1	82.53	15.95	0.49	0.18	0.85	Si + α − Al
Dot 2	70.84	14.78	12.36	2.03	0	Al_15_(Fe, Mn)_3_Si_2_
Dot 3	88.74	6.97	3.13	0.55	0.47	Al_15_(Fe, Mn)_3_Si_2_ + Si + α − Al
Dot 4	83.56	9.97	0.39	0.24	5.83	Mg_2_Si + Si + α − Al

**Table 5:** Max tension-shear load of laser-welded joints under different oscillation amplitudes.

Process Parameters		Without Oscillation	4 mm	5 mm	6 mm	7 mm
payloads/(N)	Sample-1	1484	2222	1654	2279	2616
	Sample-2	1526	2319	1789	2430	2761
	Sample-3	1606	2409	1831	2471	3044
displacement/(mm)	Sample-1	0.29	0.61	0.34	0.69	1.13
	Sample-2	0.33	0.68	0.37	0.73	1.17
	Sample-3	0.35	0.74	0.41	0.75	1.21

## Data Availability

The original contributions presented in this study are included in the article. Further inquiries can be directed to the corresponding author.
